# Arthrogryposis–renal dysfunction–cholestasis (ARC) syndrome: from molecular genetics to clinical features

**DOI:** 10.1186/s13052-014-0077-3

**Published:** 2014-09-20

**Authors:** Yaoyao Zhou, Junfeng Zhang

**Affiliations:** Department of Cardiology, No. 3 People’s Hospital, Shanghai Jiao Tong University School of Medicine, No. 280, Mohe Road, Baoshan District, 201900 Shanghai, China

**Keywords:** ARC syndrome, Arthrogryposis, Cholestasis, Renal dysfunction, *VPS33B*, *VIPAR*

## Abstract

**Abstract:**

Arthrogryposis-renal dysfunction-cholestasis (ARC) syndrome is a rare but fatal autosomal recessive multisystem disorder caused by mutations in the *VPS33B* or *VIPAR* gene. The classical presentation of ARC includes congenital joint contractures, renal tubular dysfunction, and cholestasis. Additional features include ichthyosis, central nervous system malformation, platelet anomalies, and severe failure to thrive. Diagnosis of ARC syndrome relies on clinical features, organ biopsy, and mutational analysis. However, no specific treatment currently exists for this syndrome.

**Conclusion:**

This is an overview of the latest knowledge regarding the genetic features and clinical manifestations of ARC syndrome. Greater awareness and understanding of this syndrome should allow more timely intervention with potential for improving long-term outcome.

## “What is known - what is new” (Authors’ summary)

Arthrogryposis-renal dysfunction-cholestasis (ARC) syndrome (MIM 208085), caused by mutations in the *VPS33B* or *VIPAR* gene, is a rare autosomal recessive multisystem disorder involving the liver, kidney, skin, and central nervous and musculoskeletal systems. In general, case reports of patients with ARC syndrome are not uncommon in Saudi Arabia and Pakistan, along with several sporadic cases all around the world. To help clinicians raise awareness of general clinical picture of ARC syndrome, we comprehensively characterize its major clinical presentation, namely, arthrogryposis, renal dysfunction, cholestasis, and other associated features.

Though a Leiden Open-Source Variation Database (LOVD) for ARC has been established by collating all relevant published variants observed in VPS33B and VIPAR, further analysis is still in urgent need to highlight variants that have been classified as “pathogenic” worldwide, and ultimately facilitate accurately counseling and improved disease management. Thus, we compile a total of 49 pathogenic *VPS33B* mutations and 14 pathogenic *VIPAR* mutations listed in the ARC-LOVD database to date.

All in all, this article focuses on the latest knowledge regarding both clinical and genetic features of ARC syndrome and discusses appropriate diagnosis and available treatment option currently, which gives clinicians an insight in children at risk of dying from this severe disease. Moreover, it still stands in need of future attempts at gene therapy for improvements in managing or even curing ARC syndrome.

## Introduction

**Figure 1:**
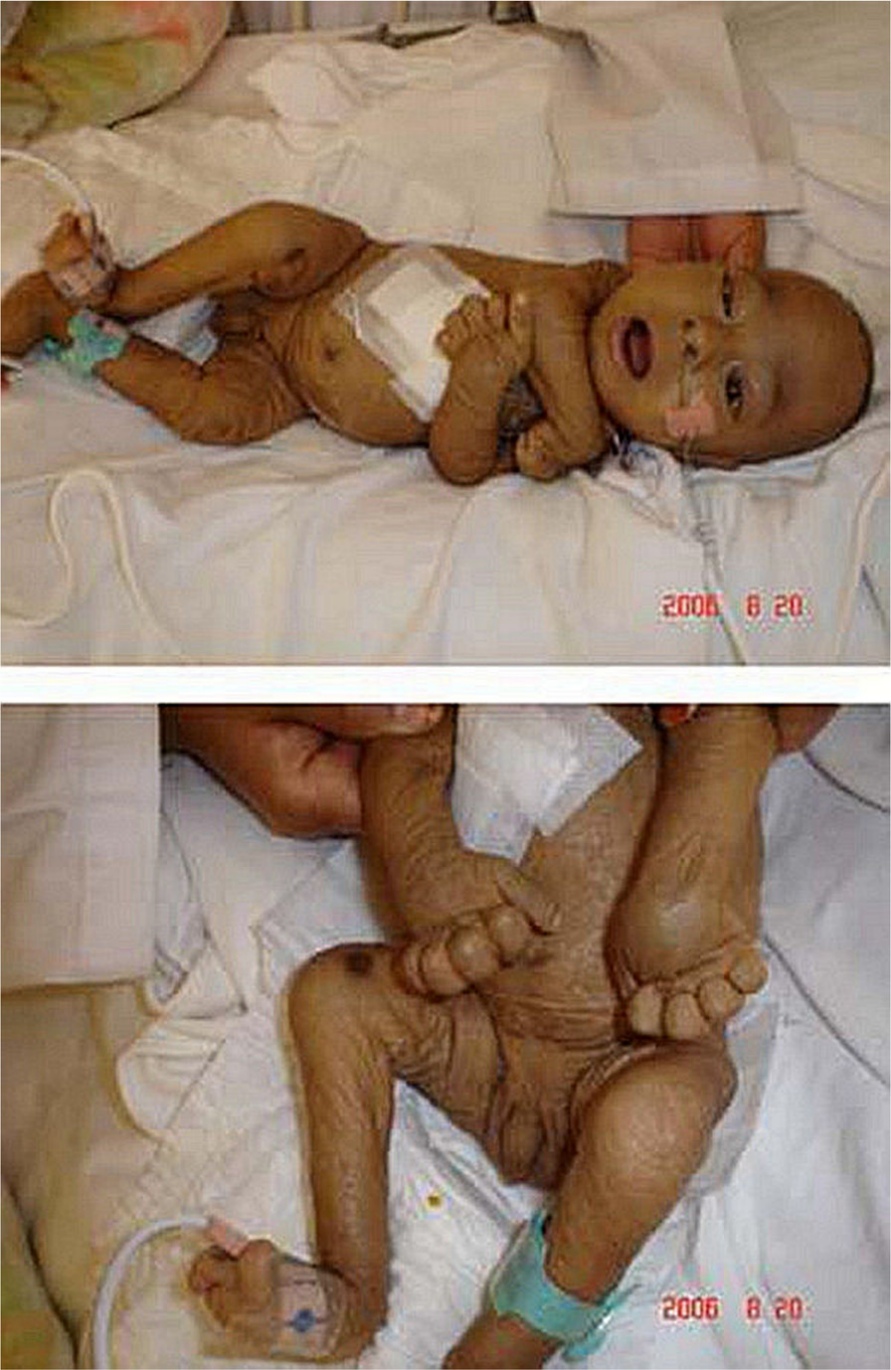
**An infant with ARC syndrome showing arthrogryposis and ichthyotic skin.** (Reproduction permission of John Wiley and Sons License, Number: 3438240519407).

Arthrogryposis–renal dysfunction–cholestasis (ARC) syndrome (MIM 208085) is a rare autosomal recessive disorder, which was first recognized in the offspring of a consanguineous marriage in 1973 [1]. As a fatal multisystem disorder, an affected child would present a series of clinical features in musculoskeletal systems, kidney, liver, and central nervous at birth. The characteristic features of ARC syndrome include arthrogryposis, renal tubular acidosis, and neonatal cholestatic jaundice (see Figure 1) [2]. These features are sometimes accompanied by additional presentations, including ichthyosis (~50%), platelet anomalies (~25%), agenesis of the corpus callosum (>20%), congenital cardiovascular anomalies (~10%), deafness, recurrent infection, and internal bleeding owing to coagulation dysfunction (see Table 1). The laboratory investigations and biopsy findings of liver or kidney could contribute to further evaluation and confirmation of ARC syndrome. Still, it may be very likely that mild or atypical symptoms at birth and during the first few weeks would lead to ignorance, misdiagnose and delayed treatment of this life-threating disorder. Consequently, the prognosis of ARC syndrome is so poor that the majority of patients fail to survive beyond the first year of life [3,4].

**Table 1:** **Clinical characteristics of patients with ARC syndrome**

**Classification**	**Clinical characteristics**
**Classical clinical feature**	Arthrogryposis
	Renal tubular dysfunction
	Neonatal cholestatic jaundice
**Additional clinical feature**	Ichthyosis
	Platelet abnormality
	Agenesis of the corpus callosum
	Congenital cardiovascular anomalies
	Deafness
	Recurrent sepsis
	Hypothyroidism
	Nephrogenic diabetes insipidus

The locus of this disorder has been mapped to chromosome 5q26.1, and germline mutations have been identified in vacuolar protein sorting 33 homolog B (*VPS33B*; MIM 608552) and VPS33B-interacting protein, apical–basolateral polarity regulator (*VIPAR*; MIM 613401) [3,5,6]. *VPS33B* encodes a 617-amino-acid protein that is a homolog of yeast Vps33p, a class C vacuolar protein sorting (vps) protein. Vps33p, along with other class C vps proteins, comprise the two multiprotein complexes, homotypic protein sorting (HOPS) and class C core vacuole/endosome tethering (CORVET), to play an essential role in intracellular vesicular trafficking pathways [7]. VPS33B is a member of the Sec1/Munc18 family proteins, which interact with soluble NSF attachment protein receptors (SNAREs). SNAREs are involved in a variety of processes—including vesicular exocytosis, synaptic transmission, and general secretion—by facilitating vesicle targeting and fusion [8]. Once mutations develop in the human *VPS33B* gene, the interaction between the expressed mutant protein and the SNARE protein at the late endosomal stage may be impeded and lead to abnormal localization or accumulation of plasma proteins in polarized cells, providing partial insights into the nature of the molecular pathophysiology of ARC syndrome.

*VPS33B* mutations are detectable in approximately 75% of patients with a clinical diagnosis of ARC syndrome [9]. Apart from locus heterogeneity or failure to detect mutation by direct sequencing analysis, another causative gene of the ARC syndrome, *VIPAR* (also called C14ORF133), was subsequently identified by combining functional and genetic approaches [6]. VIPAR consists of a golgin A5 domain and shares significant homology with the C-terminal region of Vps16, which exhibits pleiotropic effects in polarity and apical membrane protein restriction through the formation of VPS33B-VIPAR complexes [10]. The role of VPS33B-VIPAR complexes are suggested to involve the RAB11A-dependent apical recycling pathway and transcriptional regulation of adherent proteins such as E-cadherin, which ensures normal cellular structure with apical basolateral polarity [6]. It is noteworthy that further research has confirmed the role of epidermal growth factor (EGF) stimulation in the interactions between SPE-39—the *Caenorhabditis elegans* ortholog of VIPAR—and Vps33B by tyrosine phosphorylation and ubiquitination of SPE-39 [11-13]. Alternatively, the apical membrane protein was observed to be misrecruited to basolateral membranes and into the late endosomes and lysosomes in knockdown/knockout studies of *VPS33B* or *VIPAR* [14]. Subsequently, abnormal organelle biogenesis may hinder the generation and maintenance of tissue structures, such as bile ducts and renal tubules, ultimately resulting in cholestasis and abnormal urine. These proteins are found at various locations throughout the body, including the skeletal muscles, kidneys, liver, skin, heart, lungs, and brain, which explains the multisystemic symptoms that are characteristic of the ARC clinical phenotype [5,6,15].

## Clinical presentation of ARC syndrome

### *Arthrogryposis*

Arthrogryposis is a primary symptom of ARC syndrome and presents with a spectrum of manifestations, including muscle atrophy, radial deviation of the wrist, dislocation of both hip joints, flexion contracture of the knee joints, and calcaneovalgus [2]. Musculoskeletal abnormalities observed during the first few weeks of life are not generally evident—or perhaps they are simply absent or atypical in certain instances of *VPS33B* mutations, such as 971delA/K324fs [16,17]. The pathogenesis characteristic of ARC syndrome is primarily degeneration of anterior motor neurons, whereas the severity of arthrogryposis may be traced to placental insufficiency during pregnancy with oligohydramnios in the mother and growth restriction of the fetus. In addition, osteopenia and pathological fractures in ARC syndrome are related to impaired reabsorption linked to renal tubular and secondary hyperparathyroidism. Nevertheless, fractures and osteopenia are due to decreased or absent fetal movements in other kind of congenital arthrogryposis such as Bruck syndrome [18]. In case of osteopenia and fractures with arthrogryposis at birth, it is also suggested that ARC syndrome should be included in the differential diagnosis. Especial attention should be paid to the patients associated with other clinical features, such as renal tubular dysfunction, and cholestasis.

### Renal tubular dysfunction

Renal tubular dysfunction manifests in the form of Fanconi syndrome, with symptoms including renal tubular acidosis, nephrogenic diabetes insipidus, glucosuria, aminoaciduria, and phosphaturia [19,20]. During episodes of intercurrent illness, renal tubular acidosis may be notably exacerbated, which is symptomatic of renal tubular calcification and degeneration. Renal ultrasonography may be suggestive of nephrocalcinosis or a small dysplastic kidney, accompanied by inflammatory reaction of the renal interstitium and focus, sclerosis of some glomeruli, and tubular distortion and degeneration, per the results of renal biopsy [3,16].

### Neonatal cholestatic jaundice

The third primary feature of ARC syndrome is neonatal cholestatic jaundice, concurrent with hepatomegaly, which is the most common characteristic of ARC syndrome. The presentation of neonatal cholestatic jaundice in ARC syndrome is distinct from the other clinical presentations of neonatal cholestatic jaundice; patients who have ARC syndrome and develop neonatal cholestatic jaundice typically present with no biliary obstruction, have consistently low γ-glutamyl transpeptidase (γGT) levels, and have normal or slightly elevated aspartate aminotransferase (AST) and alanine aminotransferase (ALT) levels, although they have jaundice and liver cell dysfunction [17]. Indeed, it is well accepted that low GGT cholestasis is a differential feature of ARC syndrome. It is even recommended that patients with low-GGT conjugated hyperbilirubinemia associated with ichthyosis, deafness, platelet dysfunction and central nervous system malformation should be related to VPS33B disease [17].Furthermore, liver biopsies suggest signs of paucity of bile ducts, giant cell transformation, bile plug or lipofuscin deposition, and portal fibrosis in these cases, which could exclude biliary atresia specifically [3,21,22].

### Other associated features of ARC syndrome

Additional clinical symptoms of ARC syndrome principally include ichthyosis, abnormal platelet count and function, secondary infection, and cardiovascular anomalies [3,9]. Most patients are affected with ichthyosis (a heterogeneous family of skin disorders) stemming from defects in the SNARE protein, which participates in secretion and function in epidermal cohesion and waterproofing of lamellar granules [23,24]. Despite the fact that abnormal lamellar granule secretion exist in both ARC syndrome and cerebral dysgenesis–neuropathy–ichthyosis–keratoderma (CEDNIK) syndrome, lamellar granule internal structure is normal in ARC syndrome while it is abnormal in CEDNIK syndrome. An additional cause of ichthyosis in ARC syndrome is the lack of absorption of free fatty acids, which are critical for epidermal differentiation [25]. Furthermore, skin biopsy may indicate the presence of mild hyperkeratosis without parakeratosis. Self-limiting intra-abdominal hemorrhage often occurs in patients with ARC, in the absence of abnormal platelet morphology; therefore, normal routine platelet analysis cannot assess the risk of bleeding in ARC syndrome [9]. Despite the increased number of β-granules, similar to Grey platelet syndrome, platelets from patients with ARC syndrome develop abnormal biosynthesis and function of α-granules, which are essential for platelet aggregation, thrombogenesis, inflammation, and tumorigenesis [26,27]. Studies have demonstrated that a VPS33B-VPS16B complex participates in α-granule formation, since this complex was tracked with transport vesicles destined toward the development of mature α-granules [28,29]. Moreover, patients with ARC syndrome primarily have recurrent episodes of secondary infection coupled with hyperpyrexia and chronic diarrhea, although their immunological profiles are found to be within normal limits. It has been recently demonstrated that a profound defect in phagosome-lysosome fusion caused by Vps16B/Vps33B dysfunction may render patients with ARC syndrome increasingly sensitive to infections by nonpathogenic microbes [30]. Interestingly, bacterial endocarditis subject to recurrent infection has also been reported to originate from congenital cardiovascular anomalies, other than the structural abnormalities caused by defects in vesicular trafficking [3].

## Genetic background

To gain an insight into worldwide genetic epidemiology and provide easy access to updated resources for researchers and clinicians, a Leiden Open-Source Variation Database (LOVD) for ARC (https://grenada.lumc.nl/LOVD2/ARC) was established in 2011 by collating all relevant published variants observed in *VPS33B* and *VIPAR* [31]. As for March 2014, this online locus-specific ARC database has compiled a total of 299 unique variants in *VPS33B* and 34 unique variants in *VIPAR,* of which sequence mutations are basically classified as “pathogenic,” “probably pathogenic,” “no known pathogenicity,” “probably no pathogenicity,” and “effect unknown,” according to their projected effect on the protein and the clinical phenotype. To date, the database includes 49 published variants in *VPS33B* and 14 published variants in *VIPAR* worldwide that have been classified as “pathogenic.” Regarding *VPS33B*, most identified variants were substitutions (n = 34; 19 splice site, 13 nonsense, and two missense mutations), apart from deletions (n = 11), duplications (n = 2), insertions (n = 1), and indels (n = 1). It is noteworthy that three variants were prominent on account of their relative prevalence: c.403 + 2 T > A, c.1312C > T, and c.1519C > T (see Table 2). Most “pathogenic” variants in *VIPAR* are substitutions (n = 11; 8 nonsense, two missense, and one splice site mutation). Additionally, two deletions were present, of which two recurrent variants existed: c.658C > T and c.808C > T (see Table 3). This information is available in the ARC–LOVD database to inform clinicians and patient families on current prognoses, advances, and clinical course of ARC pathogenesis, ultimately contributing to accurately counseling and improved disease management.

**Table 2:** **Pathogenic** ***VPS33B*** **mutations listed in the ARC-LOVD database**

**Database ID**	**Exon**	**DNA change**	**Status**	**Protein change**	**Ethnicity**	**Reference**
VPS33B_00235	1-23	c.(?_-354)_(*431 + d127_?)del	Het	p.(0?)	Hispanic	[14]
VPS33B_00232	△4	c.240-577_290-156del	Het	p.(Leu81Serfs*5)	South American	[14]
VPS33B_00221	1	c.67C > T	Het	p.(Arg23*)	-	[14]
VPS33B_00001	1	c.89 T > C	Hom	p.(Leu30Pro)	Pakistani	[3]
VPS33B_00223	1i	c.97-2A > C	Hom	p.(?)	-	[14]
VPS33B_00002	2	c.151C > T	Het	p.(Arg51*)	French	[8]
VPS33B_00011	2i	c.177 + 1G > A	Hom	p.(?)	Italian	[3]
VPS33B_00231	2i	c.178-2A > C	Hom	p.(?)	Turkish	[14]
VPS33B_00224	2i	c.178-1G > C	Hom	p.(?)	Pakistani	[14]
VPS33B_00233	3i	c.240-1G > C	Hom	p.(?)	-	[14]
VPS33B_00003	4	c.277C > T	Het	p.(Arg93*)	South American	[8]
VPS33B_00004	5	c.319C > T	Het	p.(Arg107*)	Scottish	[8]
VPS33B_00005	5	c.352C > T	Hom	p.(Gln118*)	Turkish	[7]
VPS33B_00023	5	c.350del	Hom	p.(Pro117Leufs*20)	Saudi Arabia	[3]
VPS33B_00024	6	c.369_370del	Het	p.(Cys123*)	South American	[8]
VPS33B_00013	6i	c.403 + 1G > T	Het	p.(?)	Scottish	[8]
VPS33B_00012	6i	c.403 + 1G > A	Het	p.(?)	Israel	[3]
VPS33B_00014	6i	c.403 + 2 T > A	Het	p.(?)	Korean	[7]
VPS33B_00025	7	c.436_445del	Het	p.(Leu146Metfs*5)	French	[8]
VPS33B_00015	7i	c.498 + 1G > A	Het	p.(?)	Swedish	[4]
VPS33B_00026	8	c.558_559del	Het	p.(Tyr187Trpfs*18)	Italian	[8]
VPS33B_00006	9	c.661C > T	Het	p.(Arg221*)	Korean	[21]
VPS33B_00016	9i	c.701-1G > C	Hom	p.(?)	Israel	[25]
VPS33B_00017	9i	c.700 + 1G > A	Het	p.(?)	Saudi Arabia	[15]
VPS33B_00225	10	c.711del	Het	p.(Phe237Leufs*2)	Pakistani	[14]
VPS33B_00007	10	c.728C > T	Het	p.(Ser243Phe)	Korean	[7]
VPS33B_00027	10	c.740_741del	Het	p.(Tyr247*)	Korean	[7]
VPS33B_00226	11i	c.853-3C > G	Hom	p.(?)	Turkish	[14]
VPS33B_00019	11i	c.853-2A > G	Het	p.(?)	Portuguese	[3]
VPS33B_00018	12i	c.940-1G > A	Het	p.(?)	French	[8]
VPS33B_00028	13	c.971del	Hom	p.(Lys324Argfs*11	Pakistani	[17]
VPS33B_00227	13i	c.1030 + 5G > T	Hom	p.(?)	Saudi Arabia	[14]
VPS33B_00029	16	c.1208del	Het	p.(Leu403Cysfs*8)	Tahitian	[8]
VPS33B_00230	16i	c.1225 + 5G > C	Het	p.(?)	South American	[14]
VPS33B_00033	17	c.1235_1236delCCinsG	Hom	p.(Pro412Argfs*7)	Polish	[7]
VPS33B_00229	17	c.1261_1262del	Het	p.(Gln421Valfs*8)	South American	[14]
VPS33B_00008	18	c.1312C > T	Hom	p.(Arg438*)	Pakistani	[3]
VPS33B_00008	18	c.1312C > T	Het	p.(Arg438*)	Saudi Arabia	[15]
VPS33B_00008	18	c.1312C > T	Het	p.(Arg438*)	Pakistani	[14]
VPS33B_00219	18i	c.1406-2A > G	Hom	p.(?)	Saudi Arabia	[3]
VPS33B_00220	18i	c.1406-1G > C	Hom	p.(?)	Turkish	[8]
VPS33B_00228	20	c.1498G > T	Hom	p.(Glu500*)	Hispanic	[14]
VPS33B_00030	20	c.1509dupG	Het	p.(Lys504Glufs*23)	Korean	[7,21]
VPS33B_00009	20	c.1519C > T	Het/Hom	p.(Arg507*)	Portuguese	[3,18]
VPS33B_00218	20	c.1519C > T	Het	p.(Arg507*)	Korean	[21]
VPS33B_00031	20	c.1576_1577insT	Hom	p.(Glu526Valfs*13)	Polish	[7]
VPS33B_00010	21	c.1594C > T	Hom	p.(Arg532*)	Pakistani	[3]
VPS33B_00234	21i	c.1657 + 1G > A	Hom	p.(?)	Italian	[14]
VPS33B_00032	23	c.1803dupA	Het	p.(Val602Serfs*13)	Korean	[7]

Del, deletion; Fs, frameshift; i, intron; ∗, stop; △, whole exon deletion; Het, heterozygous; Hom, homozygous.

p.(?), effect of the variant on the protein is unknown.

p.(0?), no protein product is predicted.

**Table 3:** **Pathogenic** ***VIPAR*** **mutations listed in ARC-LOVD database**

**Database ID**	**Exon**	**DNA change**	**status**	**Protein Change**	**Ethnicity**	**Reference**
VIPAR_00001	1	c.2 T > G	Hom	p.(Met1Arg)	Turkish	[4]
VIPAR_00021	6	c.463_464del	Het	p.(Trp155Glufs*4)	Caucasian	[14]
VIPAR_00022	6	c.484C > T	Het	p.(Arg162*)	Caucasian	[14]
VIPAR_00002	7	c.535C > T	Hom	p.(Gln179*)	Turkish	[4]
VIPAR_00023	9	c.638 T > C	Het	p.(Leu213Pro)	-	[14]
VIPAR_00003	9	c.658C > T	Hom	p.(Arg220*)	Italian	[4]
VIPAR_00003	9	c.658C > T	Het	p.(Arg220*)	Turkish	[4]
VIPAR_00007	10	c.749_753del	Hom	p.(Thr250Argfs*17)	Croatian	[4]
VIPAR_00004	11	c.808C > T	Hom	p.(Arg270*)	Israel	[4,14]
VIPAR_00020	11i	c.837-1G > T	Hom	p.(?)	-	[14]
VIPAR_00005	12	c.871C > T	Het	p.(Gln291*)	Turkish	[4]
VIPAR_00019	13	c.1021 T > C	Hom	p.(Cys341Arg)	Pakistani	[14]
VIPAR_00006	17	c.1273C > T	Hom	p.(Gln425*)	Turkish	[4]

Del, deletion; Fs, frameshift; i, intron; ∗, stop; △, whole exon deletion; Het, heterozygous; Hom, homozygous.

p.(?), effect of the variant on the protein is unknown.

p.(0?), no protein product is predicted.

## Diagnostic clues and workup

In general, case reports of patients with ARC syndrome are not uncommon in Saudi Arabia and Pakistan, where rates of consanguinity are high, whereas several sporadic case studies have been reported in Turkey, North Africa, Italy, Portugal, and Asia [5,31-34]. Despite these reports, the prevalence of ARC syndrome has not been accurately defined and is subject to potential underestimation because of lack of a broad clinical picture and of early recognition of this rare disease. It is worth mentioning that the relative incidence rate ratio of ARC was suggested to be 1/7 that of biliary atresia in 90 patients with neonatal cholestasis (95% CI 0.33 ~ 0.06) [21,22,35].

ARC syndrome is a life-threatening autosomal recessive multisystem disorder, and its early diagnosis is of vital importance for the development of an appropriate therapeutic regimen. Currently, clinical diagnosis of ARC syndrome consists of identifying the triad conditions of arthrogryposis, renal tubular acidosis, and neonatal cholestatic jaundice with low γGT activity, combined with pathologic confirmation. However, the majority of patients are vulnerable to coagulation defects; in other words, kidney or liver biopsies result in a risk of fatal bleeding (>50%). Still, similar clinical and laboratory findings could be observed both in ARC syndrome and progressive familial intrahepatic cholestasis. Therefore, clinical presentations along with *VPS33B* and *VIPAR* sequencing analyses constitute an apparently safer diagnostic procedure. Currently, there are limitations in mutational analysis, such as the long duration required for analysis and the potential for false negatives, analysis of VPS33B protein expression in skin fibroblasts and platelet morphology in peripheral blood smears are two alternative techniques that have been proposed as valuable tools for diagnostic screening examinations [9,36].

## Treatment options and prognosis

No specific treatment for ARC syndrome currently exists; rather, supportive care—including fluid infusion, anti-infection, supplement with ursodeoxycholic acid, fat-soluble vitamins, calcium glubionate, L-thyroxine and phosphate—is administered to patients for improving the quality of life. Nevertheless, some patients with joint contractures, congenital hip dislocation, and vertical talus are in need of immediate orthopedic intervention due to delayed diagnosis. Nevertheless, aggressive orthopedic management is still not recommended, since poor general status and low survival rates may affect the outcome of the surgery [37]. In cases of ARC syndrome that failed to respond to medical therapy, it is also advisable to consider liver transplant to ameliorate severe cholestasis and intractable pruritus. It is reported that an Iranian boy underwent a liver transplant have made a recovery. Specifically, the pruritus immediately improved after the surgery, and his scaly skin was also normal in 6 months. Moreover, the patient stayed in good condition without any complications or rejection during more than 5 years’ follow-up [38].

As a lethal multisystem disorder, prognosis of ARC syndrome is particularly poor. Most patients succumb within the first year of life after developing recurrent infection, severe hydropenia, acidosis, or internal hemorrhaging, except for a few patients who have ARC syndrome but retain partial function of *VPS33B* [22]*.*

## Conclusion

No specific treatment currently exists for ARC syndrome. Comprehensive analysis of family history, classical clinical presentations, biopsy of the liver or kidney, and/or genetic mutational analysis may not only facilitate accurate diagnosis and the development of appropriately tailored treatment at an early stage but also provide genetic counseling and prenatal or preimplantation genetic diagnosis for the affected families. With the continued progress of molecular genetics and medical technologies, future attempts at gene therapy may yield improvements in managing or even curing ARC syndrome.

### Consent

Written informed consent was obtained from the patient’s guardian/parent/next of kin for the publication of this report and any accompanying images.

## Abbreviations

ALT: alanine aminotransferase
AST: Aspartate aminotransferase
ARC: ARTHROGRYPOSIS-renal dysfunction-cholestasis
CEDNIK: Cerebral dysgenesis–neuropathy–ichthyosis–keratoderma
CORVET: Class C core vacuole/endosome tethering
EGF: Epidermal growth factor
γGT: γ-Glutamyl transpeptidase
HOPS: Homotypic protein sorting
LOVD: Leiden open-source variation database
SNAREs: Soluble NSF attachment protein receptors
*VPS33B*: Vacuolar protein sorting 33 homolog B
*VIPAR*: VPS33B-interacting protein, apical–basolateral polarity regulator
